# Multi-drug resistant toxigenic *Vibrio cholerae* O1 is persistent in water sources in New Bell-Douala, Cameroon

**DOI:** 10.1186/1471-2334-13-366

**Published:** 2013-08-07

**Authors:** Jane-Francis Tatah Kihla Akoachere, Thomas Njinuwoh Masalla, Henry Akum Njom

**Affiliations:** 1 Department of Microbiology and Parasitology, Faculty of Science, University of Buea, Buea, Cameroon; 2 Laboratory for Emerging Infectious Diseases, Faculty of Science, University of Buea, Buea, Cameroon

**Keywords:** *Vibrio cholerae* O1, Cholera, Virulence genes, Antibiotic susceptibility, Multidrug resistance, Environmental factors, Cameroon

## Abstract

**Background:**

Cholera has been endemic in Douala, since 1971 when it was first recorded in Cameroon. Outbreaks have often started in slum areas of the city including New Bell. Despite the devastating nature of outbreaks, always resulting in high mortality and morbidity, a paucity of information exists on the reservoirs of the causative agent, *V. cholerae,* and factors maintaining its persistence. This has complicated disease prevention, resulting in frequent outbreaks of cholera. We investigated water sources in New Bell for contamination with *V. cholerae* O1 with pathogenic potential, to highlight their role in disease transmission. Antibiotic susceptibility pattern of isolates and the environmental factors maintaining its persistence were investigated.

**Method:**

Water samples from various sources (taps, dug wells, streams) were analyzed for contamination with *V. cholerae* O1 using standard methods. Antibiotic susceptibility was determined by disc diffusion method. Pathogenic potential of isolates was determined by analyzing for genes for cholera toxin (*ctx*), toxin co-regulated pilus (*tcpA*), and zonula occludens toxin (*zot*) by PCR. Physico-chemical characteristics of water (pH, temperature and salinity) were investigated using standard methods. The Spearman’s Rank correlation was used to analyze the relationship between physico-chemical factors and the occurrence of *V. cholerae* O1*.* Differences were considered significant at P≤0.05.

**Results:**

Twenty-five *V. cholerae* O1 strains were isolated from stream and well samples in both dry and rainy seasons. Twenty-three (92%) isolates were multidrug resistant. All isolates had genes for at least one virulence factor. Cholera toxin gene was detected in 7 isolates. Of the 15 isolates positive for *tcpA* gene, two had Classical type *tcpA* while 13 had *tcpA* El Tor. All *tcpA* Classical positive isolates were positive for *ctx* gene. Isolates were grouped into nine genotypes based on the genes analyzed. pH and salinity significantly correlated with isolation of *V. cholerae* O1.

**Conclusion:**

Multidrug resistant *Vibrio cholerae* O1 with pathogenic potential is present in some wells and streams in study area. pH and salinity are among the factors maintaining the persistence of the organism. Findings indicate an urgent need for potable water supply in study area and in addition, regular disinfection of water from contaminated sources to prevent outbreak of cholera.

## Background

Toxigenic *Vibrio cholerae* O1 and O139 are the causative agents of cholera, an epidemic and life threatening diarrheal disease, which has been a public health concern in most developing countries. Cholera is associated with poor socio-economic conditions, rudimentary sanitary systems and public hygiene, and inadequate supply of potable water. In crowded urban slum and rural areas, epidemics are characterized by high morbidity and mortality. Although *V. cholerae* is a human pathogen, it is autochthonous to the aquatic environment where it persists in the absence of a human host [1] and exists as either a free-living bacterium or in association with zooplankton. Irrespective of their toxin-producing ability, *V. cholerae* O1 rarely occurs in natural aquatic environments. Toxigenic strains are rarely isolated from surface water during interepidemic periods. Transmission usually results from drinking contaminated water [2] or eating inadequately cooked foods that have been washed or prepared with contaminated water [3].

The pathogenicity of *V. cholerae* O1 rests on its ability to express virulence factors like a potent enterotoxin, cholera toxin (CT), and the colonization factor (TCP). These major virulence factors are present in clusters within 2 regions in the *V. cholerae* chromosome: the CTX genetic element which has been reported to comprise the genome of a filamentous phage (CTXΦ) [4]; the *Vibrio cholerae* pathogenicity Island (VPI) that encodes a toxin co-regulated pilus (TCP), a type IV pilus that functions in colonization and acts as a receptor for CTXΦ. Since CTXΦ is a transferrable phage and encodes the cholera toxin [5], acquisition and expression of CT should precede infection by CTXΦ. It is generally believed that environmental strains do not produce the cholera toxin gene as such lack the potential to produce epidemic cholera. Other factors associated with enteropathogenicity include hemolysin (*hlyA*), heat stable enterotoxin (stn/sto), hemagglutinins, neuraminidase, outer membrane protein, shiga-like toxin (stx), a ToxR regulatory protein and the zonula occludens toxin (zot) [6,7]. Genes that code for cholera toxin (*ctx*) and toxin co-regulated pilus (*tcp*) are presumed to be exclusively associated with the toxigenic *V. cholerae* strains and are acquired by the bacterium from the aquatic environment [8]. Thus, the aquatic environment may play an important role in the ecology, transmission, and epidemiology of *V. cholerae*. Detection of *V. cholerae* in water is therefore important for disease prevention and control.

Cholera could result in high fatality rates if disease is not properly managed. Although rehydration plays a pivotal role in reducing mortality, antibiotics have been used to reduce the shedding of the organism (thereby reducing spread of the disease), treating severe illness (by reducing volume of diarrhea), and also to reduce duration of disease and hospitalisation. However, the extensive use of antibiotics in therapy and prophylaxis has resulted in the emergence of drug resistant strains in cholera endemic regions [9,10] limiting their use in empiric treatment. This necessitates surveillance of antibiotic susceptibility pattern of the organism not only from clinical isolates but also from environmental isolates in endemic regions as the environment could serve as a reservoir for resistant strains.

In cholera endemic regions, several studies have reported a seasonal pattern of occurrence of *V. cholerae* and cholera [11-13]. This seasonal fluctuation has been attributed to changing environmental factors. Temperature, pH, salinity and nutrient concentration are among the environmental factors shown by field studies to influence the occurrence of the organism [14]. Since the epidemiology of cholera is closely linked to the ecology of *V. cholerae* in the environment, an understanding of the environmental factors that support its persistence and multiplication is crucial for public health protection.

Between 2000 and 2005, Cameroon recorded the highest mean case-fatality rate (10.2%) of cholera in Africa [15] with most outbreaks occurring in Douala, Cameroon’s biggest port city and economic capital. Outbreaks have usually started in the densely populated slums with inadequate sanitation facilities, potable water supply and poor hygiene practices. Despite the regular occurrence of the disease (an indication of persistent presence of *V. cholerae* in the environment), the emergence of drug resistant strains of *V. cholerae* in Douala [9,16] and devastative nature of outbreaks, there still exists a scarcity of data on the reservoirs of the organisms and environmental factors maintaining its persistence in Douala. Inadequate information has complicated disease prediction, prevention and control and has contributed to frequent outbreaks of cholera in Douala.

*V. cholerae* has been reported to contain a distinct class of integrons which permit it to acquire open reading frames and convert them to new functional genes. This implies that not only non-toxigenic strains can acquire virulence genes from the environment but acquisition of antibiotic resistance genes is also possible [17]. Given that the massive use of antibiotics in prophylaxis during previous cholera outbreaks in Douala resulted in the selection of multidrug resistant strains of *V. cholerae*[9,16] current data on antibiotic susceptibility pattern of isolates will update knowledge on appropriate antibiotics for use in empiric treatment in case of an outbreak.

Among the slum areas of Douala, New Bell has been severely affected by cholera epidemics. Outbreaks of the disease have often started from New Bell and this locality has always recorded the highest number of cases and deaths. This study was carried out to isolate and determine the antibiotic susceptibility pattern of *V. cholerae* O1 with pathogenic potential from water sources in New Bell and evaluate the physico-chemical factors that maintain its persistence. Findings of this study will not only provoke policies that will drive Cameroon towards the achievement of the Millennium Development Goals target for drinking water but will be of great value in the prevention and control of cholera in study area.

## Methods

### Study site

**Figure 1:**
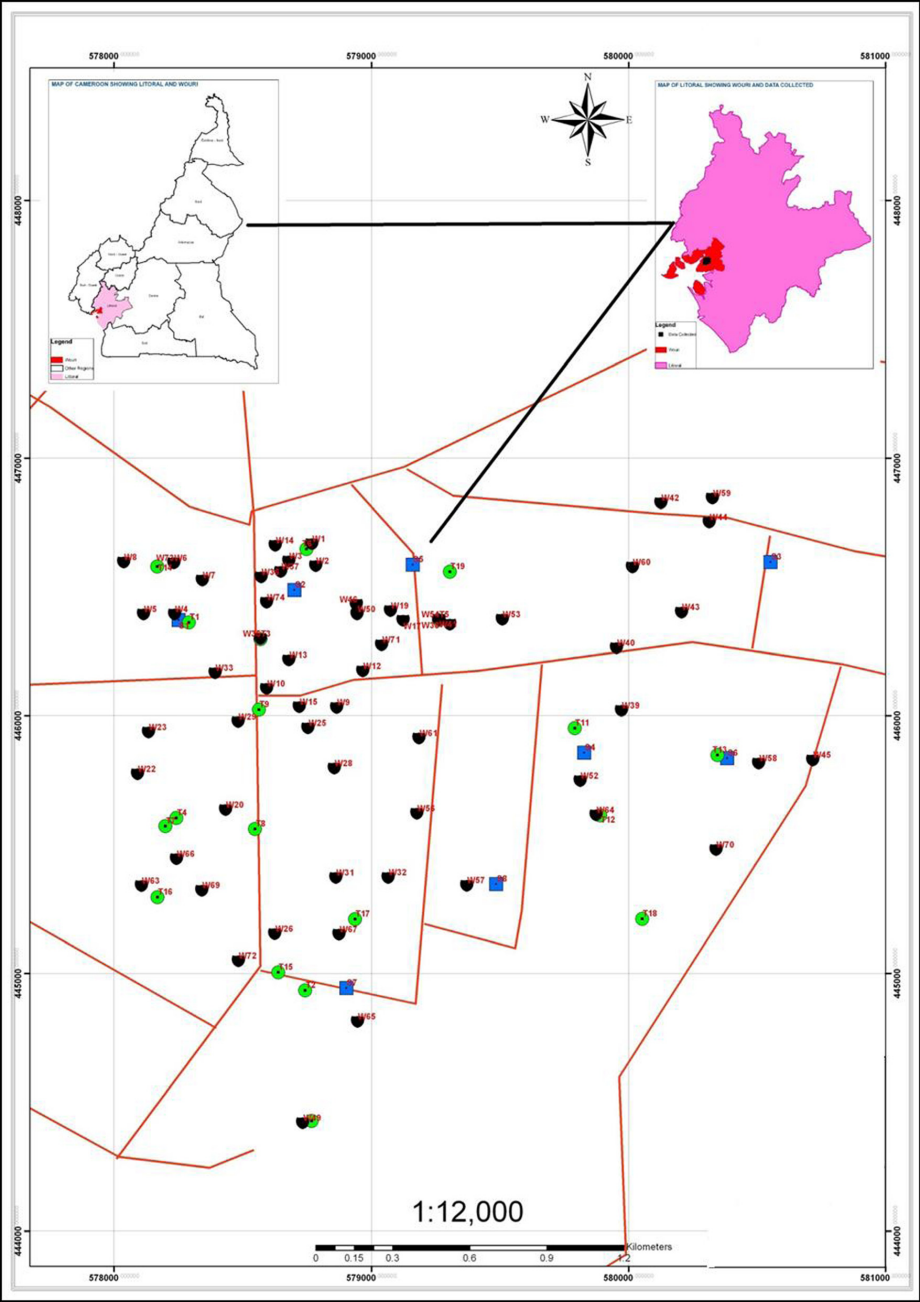
**Map of New Bell showing sampling stations.** “Green circle symbol” Stream samples, “black semi-circle”Tap samples, “blue square symbol” Well samples, “red lines” Roads.

This study was carried out in New Bell (Figure 1), a densely populated neighbourhood in the city of Douala. Douala, is located along the coastal plain of Cameroon and has an equatorial climate with two seasons; the dry season which begins in November and ends in April with January and February as the hottest months [18], the rainy season begins from May to October, August and September having the highest rainfall. However, there is occasional rainfall during the dry season. The New Bell district is one of the areas that have been severely affected by cholera outbreaks. During the 2010–2011 epidemic, the highest number of cases and deaths in the Littoral region were from this locality [19]. Potable water scarcity is an acute problem in New Bell. Since the water table is high, inhabitants rely on shallow wells for water used for drinking and other purposes. Streams in New Bell are used for bathing, washing, recreation and irrigation.

### Study design

Samples were collected from streams, shallow-wells and taps (communal and private standpipes) in New Bell during the dry and rainy seasons and analyzed for *V. cholerae* O1. The public health significance of isolates was determined by analyzing their antibiotic susceptibility pattern and also testing for the presence of genes for virulence factors cholera toxin (*ctx*), toxin co-regulated pilus (*tcpA*) and zonular occludens toxin (*zot*). The relationship between some environmental factors (temperature, pH and salinity) and the occurrence of *Vibrio cholerae* O1 was studied.

### Sample collection

From March 2010 to January 2011, 300 water samples were aseptically collected from randomly selected streams and house hold sources including shallow wells and taps in New Bell (Figure 1) into sterile glass bottles (500 mL). A total of 8 streams, 74 wells, and 19 taps were sampled. To collect well or stream sample, a sterile wedge was tied to the sample container and immersed into water until container was full. Stream samples were collected at a depth of about 30 cm and 2-3 m away from the bank. Prior to collection of tap water samples, the tap opening was cleaned with 70% alcohol, and water allowed to flow for 3 minutes to flush out any contaminants. Samples were transported at 4°C from site to the laboratory.

### Isolation and characterization of *V. cholerae*

The method described by *Islam et al.*[20] was employed with slight modification. Ten millilitres (10 mL) of sample enriched in 5 mL of triple strength alkaline peptone water (Liofilchem s.r.i. Bacteriology Products, Italy), pH 8.4 overnight at 37°C. A loop full of enrichment culture was picked just beneath the surface of broth and streaked onto thiosulfate citrate bile salt sucrose (TCBS) agar (Liofilchem s.r.I. Bacteriology Products, Italy) plates and incubated at 37°C for 18–24 hours. Presumptive colonies (yellow, measuring 2-4 mm) were sub cultured on brain heart infusion agar to obtain pure cultures. Gram negative, curved and motile rods that were catalase positive, oxidase positive and fermented glucose (but not lactose or sucrose) without gas production or blackening of Kliger Iron Agar medium (Laboratorios Conda, S.A, Spain) were subjected to further biochemical characterization using the API 20E kit (BioMerieux SA, France). Biochemically confirmed *Vibrio cholerae* were serotyped by slide agglutination as described by CDC [21] using *V. cholerae* O1 polyvalent antiserum (Remel Europe Ltd, UK). Isolates that did not show agglutination with polyvalent O1 antiserum were tested with O139 antiserum (Remel Europe Ltd, UK).

### Antimicrobial susceptibility testing (AST)

Susceptibility of isolates to antimicrobial agents was assessed by the disc diffusion (Kirby-Bauer) technique as described by the National Committee for Clinical Laboratory Standards (presently called the Clinical and Laboratory Standards Institute) [22]. A small inoculum of each bacterial isolate was emulsified in 3mL of sterile normal saline in Bijou bottles and the density was compared with a barium chloride standard (0.5 Mcfarland). A sterile cotton wool was dipped into the standardized suspension of bacterial culture and used to evenly inoculate Mueller-Hinton plates (Biotec, England), and the plates were allowed to dry. Antibiotic discs (Oxoid, Basingstoke, England) with the following drug concentrations: tetracycline (30 μg), doxycycline (30 μg), amoxicillin (30 μg), ampicllin (30 μg), trimethroprim-sulfamethoxazole (cotrimoxazole) (25 μg), ciprofloxacin (5 μg), chloramphenicol (30 μg) were placed on the plates. Discs were placed at least 15 mm apart and from the edge of the plates to prevent the overlapping of zones of inhibition. Plates were incubated at 37°C for 24 hours, and the diameters of zones of inhibition were compared with the recorded diameters of the control organism E. coli ATCC 25922 to determine susceptibility or resistance.

### Detection of genes for virulence factors (*ctx*A, *tcp*A, *zot*) in *V. cholerae* O1 isolates

Isolates were examined for the presence of genes for cholera toxin (*ctx*A), toxin-coregulated pilus (*tcp*A) and zonular occludens toxin (*zot*) by PCR assay. Genes for the Classical *tcpA* (*tcpA*_*CL*_) and *tcp*A El Tor (*tcpA*_*ET*_) were investigated. DNA isolation from bacterial cell cultures was done using the Qiagen DNeasy kit for Blood and Tissue isolation (Qiagen, Hilden, Germany), following the manufacturer’s instructions. Sequences of primers used were as follows: *ctx* A F: 5′−CTCAGACGGGATTTGTTAGGCACG−3′, R:5′−TCTATCTCTGTAGCCCCTATTACG−3′ which gave a product size of 302p [23]; *tcpA* El Tor F: 5′−GAAGAAGTTTGTAAAAGAAGAACAC−3′, R: 5′−GAAAGGACCTTCTTTCACGTTG−3′ with a product size of 472 bp [23] and *tcpA* Classical F:5′- CACGATAAGAAAACCGGTCCAAGAG-3′, R:5′-ACCAAATGCAACGCCGAATGGAGC-3′, with product size of 618 bp [23]. For *zot* gene, primer sequences as described by Rivera *et al.*[24] were as follows: F: 5′−TCGCTTAACGATGGCGCGTTTT−3′, R: 5′−AACCCCGTTTCACTTCTACCCA−3′and gave a product with size of 947 bp*.* Oligonucleotide primers were synthesized by Inqaba Biotechnical Industries (Pty) Ltd., South Africa.

Amplification was carried out in 25 μL volumes by adding 12.5 μL of PCR master mix (Top Taq™ Master Mix, Qiagen), 0.5 μL of each primer, 6.5 μL of sterile PCR water and 5 μL of bacterial DNA. Two negative controls with PCR water were included in each PCR run. The following amplification conditions described by Islam *et al.*[23] were used for *ctx*A, *tcp*A _ET_ and tcpA_CL_ genes: an initial denaturation at 94°C for 5 minutes, followed by a middle step of 40 cycles for 1 minute each at 94°C (denaturation), 56°C (annealing of primer) and 72°C (DNA polymerase– mediated extension) and a final extension step at 72°C for 10 minutes. For *zot* gene, PCR amplification conditions described by Rivera *et al*. [24] were used and included an initial denaturation at 94°C for 2 minutes, denaturation at 94°C for 1 minute, annealing at 60°C for 1 minute, an extension step at 72°C for 1 minute at the end of 30 cycles and a final extension step at 72°C for 10 minutes. PCR was carried out using an automated thermal cycler (Bio Rad™).

Amplification of DNA was analysed electrophoretically in 1.5% agarose gel for the *ctxA* and *tcpA* genes and 1.0% agarose for *zot* gene. A 1-kb molecular size marker (New England Biolabs Inc., USA) was used for separation of the amplicons. Gels were stained with the amplified DNA and visualized with a UV transilluminator and photographed using a Gel Documentation-XR reader (BIORAD, Hercules, CA).

### Determination of environmental parameters

Water temperature, pH and salinity were measured on site immediately after sample collection. Temperature was measured using a thermometer (Chemistry Thermometer, No 3200). pH was measured using a pH meter (HANNA Instrument HI 9811, UK). Values for salinity were obtained by measuring conductivity of water samples (μS/cm) using a conductivity meter (HANNA Instrument HI 9811, UK). Conductivity values were then converted to salinity (ppt).

### Data analyses

Statistical Package for Social Science (SPSS) (version 16.0) was used to analyze data. The Chi Square (χ^2^) test was employed to examine differences in the prevalence of *V. cholerae* O1 as well as its distribution in water sources. The Spearman’s Rank correlation was used to analyze the correlation between physico-chemical parameters and the occurrence of *V. cholerae* O1*.* Wilcoxon Signed Rank (Z) test was used to analyze the differences observed in physico-chemical measurements between the dry and the rainy seasons. Differences were considered significant at P ≤ 0.05.

## Results

### Occurrence of *V. cholerae* O1 in samples

Of the 68 isolates confirmed to be *V. cholerae,* 25 (36.8%) belonged to serogroup O1 (Table 1). These were isolated from 4 of 8 streams and 14 of 50 contaminated wells (Additional file 1). Among the streams and wells, the highest recovery rate of the organism was in S2, and W25 and W68 where 5, 3, and 2 isolates respectively were obtained. Of the 25 *Vibrio cholerae* O1 isolates obtained throughout the period of study, three were detected from March to April 2010 (Additional file 2) before the start of an epidemic in May 2010. *Vibrio cholerae* O1 was detected in all the months of our sampling period. *Vibrio cholerae* O139 was not isolated. We observed co-existence of *V. cholerae* O1 and non-O1 in all 4 contaminated streams (S1, S2, S4, and S8) and two wells: W33 and W56 (Additional file 1).

**Table 1:** **Prevalence of *V. cholerae *O1 in water samples**

**Water source**	**Number**	**Number positive**	**Number**
	**of samples**	**for *V. cholerae***	**positive for O1**
	**analyzed**	**(% Positive)**	**serogroup (%)**
Stream (N = 8)	23	17 (73.9)	8 (47.1)
Tap (N= 19)	26	1 (3.8)	0 (0)
Well (N=74)	251	50 (19.9)	17 (34.0)
**Total**	**300**	**68 (22.7)**	**25 (36.8)**

(χ^2^ = 13.578, df = 2, P = 0.001).

### Seasonal distribution of *V. cholerae* O1 in various water sources

A higher frequency of isolation of *V. cholerae* O1 was recorded in the rainy season (19/49, 38.8%) than in the dry season (6/19, 31.6%). In both seasons the highest rate of isolation was from streams (Table 2). Serogroup O1 was not detected in Tap water samples. There was no significant difference in the isolation of the organism with respect to season (χ^2^ = .305, df = 2, P = 0.581).

**Table 2:** **Seasonal distribution of *V. cholerae *O1 in various water sources**

**Season**	**Water**	**No. of samples**	**Number of**	**Serogroup**
	**sources**	**analyzed**	**isolates (%)**	**O1 (%)**
Dry	Stream	8	6 (31.6)	3 (50)
	Tap	17	0 (0.0)	0 (0.0)
	Well	95	13 (68.4)	3 (23.1)
	**Total**	**120**	**19 (100.0)**	**6 (31.6)**
Rainy	Stream	15	11 (22.5)	5 (45.5)
	Tap	9	1 (2.0)	0 (0.0)
	Well	156	37 (75.5)	14 (37.8)
	**Total**	**180**	**49 (100)**	**19 (38.8)**

(χ^2^ = 0 .305, df = 2, P = 0.581).

### Antibiotic sensitivity of *V. cholerae* O1

All isolates (100%) were susceptible to ciprofloxacin. Other potent drugs were chloramphenicol (80%) and doxycycline (88%) (Additional file 3). β-lactam antibiotics [ampicillin (8.0%) and amoxicillin (12.0%)], tetracycline (32%) and co-trimoxazole (36%) had the low susceptibilities. Twenty-three (92%) isolates were multidrug resistant (resistant to two or more antibiotics). The two isolates that did not show multidrug resistance were obtained from W12 and W68. Nine multidrug resistance patterns were detected (Table 3). Pattern SXT^R^/AML^R^/AMP^R^/TE^R^ (39.1%) was the most frequently encountered followed by AML^R^/AMP^R^/TE^R^ (17.4%) and SXT^R^/AML^R^/AMP^R^/C^R^/TE^R^ (13%).

**Table 3:** **Resistance patterns of *V. cholerae *O1 isolates**

**Resistance**	**Resistance profile**	**Number**	**Source of isolate**
**code**		**exhibiting**	
		**resistance**	
		**profile (%)**	
R1	AML^R^/AMP^R^	1 (4.3)	S2
R2	AML^R^/AMP^R^/C^R^/TE^R^	1 (4.3)	S2
R3	AML^R^/AMP^R^/TE^R^	4 (17.4)	S1, W25, W33, W35
R4	DXT^R^/AML^R^/AMP^R^	1 (4.3)	W68
R5	SXT^R^/AML^R^/AMP^R^	2 (8.7)	W41, W56
R6	SXT^R^/AML^R^/AMP^R^/C^R^	1 (4.3)	S2
R7	SXT^R^/AML^R^/AMP^R^/C^R^/TE^R^	3 (13.0)	S8, W25, W49
R8	SXT^R^/AML^R^/AMP^R^/TE^R^	9 (39.1)	S2^*^, S4, W4, W53, W60, W25, W70, W73
R9	SXT^R^/AMP^R^	1 (4.3)	W16

^*^Two isolates from S2 exhibited resistance pattern R8. Tetracycline (TE), Chloramphenicol (C), Ampicillin (AMP), Amoxicillin (AML), Cotrimoxazole (SXT), Doxycycline (DXT).

### Amplification of *ctx*A, *tcp*A, and *zot* genes in isolates

All 25 isolates were positive for at least one virulence gene (Table 4). Subunit A of the cholera toxin (*ctx*A) gene was amplified in 7 (28%) isolates. Amplification of the 472 bp *tcpA*_ET_ gene occurred in 13 isolates. The gene for *tcpA*_*CL*_ was detected in 2 isolates. These 2 isolates were positive for cholera toxin gene with one of them, in addition, being positive for the *zot* gene. *zot* gene was amplified in 15 (60%) isolates (Table 4). Two isolates were positive for all three virulence genes investigated. These organisms were isolated from streams S2 (during the rainy season) and S8 (isolated in the dry season) with the S8 isolate having *tcpA* of the Classical type (Table 4).

**Table 4:** **Virulence genes in *Vibrio cholerae *O1 isolated from wells and streams**

**S/N**	***ctx*A**	***tcp*A-El Tor**	***tcpA*-classical**	***Zot***	**Source**	**Season**
1	+	-	-	+	S2	D
2	-	+	ND	-	W33	R
3	-	-	-	+	W73	R
4	-	-	-	+	W35	R
5	-	+	ND	-	W49	R
6	-	+	ND	-	S2	R
7	-	-	-	+	W4	R
8	+	-	+	-	W68	R
9	-	+	ND	+	S2	R
10	-	+	ND	-	W25	R
11	-	-	-	+	W12	R
12	-	-	-	+	W68	R
13	+	+	ND	-	W56	R
14	+	-	-	+	S2	R
15	-	+	ND	-	W60	R
16	-	+	ND	+	S1	R
17	-	-	-	+	W25	R
18	-	+	ND	-	S4	R
19	-	-	-	+	W41	R
**20**	**+**	**+**	ND	**+**	**S2**	R
21	-	+	ND	+	W70	D
**22**	**+**	**-**	**+**	**+**	**S8**	D
23	-	+	ND	-	W53	D
24	+	-	-	-	W25	D
25	-	+	ND	+	W16	D

*ND* Not Determined, +: positive, -: negative, *D* dry season, *R* rainy season.

### Genotypes of *Vibrio cholera*e O1 isolates

Based on the genes analyzed, 9 genotypes of *Vibrio cholerae* O1 were identified. Genotypes *ctxA*^*-*^*tcpAET*^+^*zot*^-^ (28%) and *ctxA*^*-*^*tcpAET*^*-*^*zot*^+^ (28%) were the most frequently detected (Table 5). More than one genotype was detected among isolates from S2, W25, and W68. One isolate from W25 had *ctxA* as the only virulence gene.

**Table 5:** **Genotypes of *Vibrio cholera*e O1 isolates**

**Genotype**	**Source(s)**	**No. of isolates (%)**
*ctxA*^*+*^*tcpA*^*-*^_*(ET)*_*zot*^*+*^	S2*, S2	2 (8)
*ctxA*^*+*^*tcpA*^*+*^_*(CL)*_*zot*^*+*^	S8	1(4)
*ctxA*^*+*^*tcpA*^*+*^_*(ET)*_*zot*^*+*^	S2	1 (4)
*ctxA*^*+*^*tcpA*^*+*^_*(ET)*_*zot*^*-*^	W56	1 (4)
*ctxA*^*+*^*tcpA*^*+*^_*(CL)*_*zot*^*-*^	W68*	1(4)
*ctxA*^*+*^*tcpA*^*-*^_*(ET)*_*zot*^*-*^	W25*	1 (4)
*ctxA*^*-*^*tcpA*^*+*^_*(ET)*_*zot*^*-*^	S2, S4, W25, W33, W49, W53, W60.	7 (28)
*ctxA*^*-*^*tcpA*^*+*^_*(ET)*_*zot*^*+*^	S1, S2, W16, W70.	4 (16)
*ctxA*^*-*^*tcpA*^*-*^_*(ET)*_*zot*^*+*^	W4, W12, W25, W35, W41, W68, W73.	7 (28)

* Water sources from which more than one genotype of *V. cholerae* O1was isolated.

### Environmental factors

Stream samples recorded the highest temperature in both rainy (26.9°C - 31.0°C) and dry (28.4°C - 32.7°C) season. Generally, temperature values dropped in all samples during the rainy season (Additional file 4). There were no significant differences (P>0.05) in the stream (Z = −1.389) and in tap water temperatures (Z = −1.781) between the rainy and dry season. However, there was a significant difference observed in well water temperatures between the two seasons (Z = −3.301, P = 0.001).There was no correlation between temperature and occurrence of *V. cholerae* O1 in the various water sources in the dry season (Spearman’s rho = −0.069, P = 0.454) and the rainy season (Spearman’s rho = −0.019, P = 0.804).

pH values of samples ranged from slightly acidic to alkaline. The lowest (5.7) and the highest pH readings (11.3) were recorded in wells in the rainy and dry season respectively (Additional file 4). Although pH values considerably dropped during the rainy season, the pH of tap water was fairly constant with no significant difference between the two seasons (Z = −1.838, P = 0.066). There were significant differences in the pH of stream and well water between the rainy and dry season (Z = −3.748; Z = −3.531 with P < 0.05 for stream and well respectively). There was a significant correlation between pH and occurrence of *V. cholerae* O1 in water sources in the dry season (Spearman’s rho = 0.227, P = 0.013) and the rainy season (Spearman’s rho = 0.380, P = 0.0001).

Values for salinity were generally higher in the rainy season. In the dry season, the highest values were obtained from well samples (1.1 – 5.16 ppt) (Additional file 4). Highest values in the rainy season were detected in stream samples (1.62 – 11.03 ppt). There were significant differences in salinity of samples from all three water sources between the rainy and dry season (Z = −3.008; Z = −3.212; Z = −3.998 with P < 0.05 for stream, tap and well respectively). There was a significant correlation between salinity and isolation of *V. cholerae* O1 in samples from various sources in the dry season (Spearman’s rho = 0.201, P = 0.003) and in the rainy season (Spearman’s rho = 00.280, P = 0.0011).

## Discussion

Cholera continues to be a devastating disease of immense public health significance particularly in developing countries. Lack of infrastructure and economic development has made many parts of sub-Saharan Africa vulnerable to cholera, a disease associated with lack of potable water and poor sanitation. Between 2000 and 2005, Cameroon recorded the highest mean case-fatality rate (10.2%) of cholera in Africa [15] with most epidemics occurring in Douala and the northern regions of the country. Since cholera is a treatable disease, case-fatality rate reflects access to basic health care. Therefore, cholera prevention and control strategies and data that will strengthen these efforts are of great value to Cameroon’s public health.

The prevalence of *V. cholerae* O1 in samples was low. Of the 68 isolates of *V. cholerae* obtained, only 25 (36.8%) belonged serogroup O1 (Table 1, Additional file 1). Of these 25 *Vibrio cholerae* O1 isolates, three were isolated between March and April before the start of the epidemic in May 2010. The organism was detected during all the months of our study (Additional file 2). Forty-three (43) isolates belonged to the non-O1 serogroup. Previous studies [25] reported a low prevalence of *V. cholerae* O1 in water in endemic regions. Majority of environmental *V. cholerae* strains have been reported to belong to the non-O1/non-O139 serogroup [14]. Although these non-O1 strains lack epidemic or pandemic potential, some isolates have been reported to possess cholera toxin (*ctx*) gene and other virulence genes [25] present in O1 serogroup. Although we did not further investigate the non-O1 isolates for virulence determinants, their presence in our study area should be considered of public health importance and should not be ignored particularly as they have occasionally been isolated from cases of cholera-like diarrhoea [26] and from a variety of extra intestinal infections [27]. In samples where the pathogen was not recovered, it could that either the prevailing environmental conditions in their niche were unfavorable for growth of *V. cholerae* or the source was regularly disinfected (wells and taps). Under unfavorable conditions *V. cholerae* cells have been shown to exist in a viable but non-cultural (VNC) form, [28] which is believed to maintain its persistence in the environment during inter-epidemic periods. Being that under favourable climatic conditions, VNC *V. cholerae* could revert to a transmissible state, cholera control strategies in endemic areas should be encouraged even when *V. cholerae* is not detected in the environment and should include the surveillance for the viable non-culturable state of the organism. The presence of viable but non-culturable cells was not investigated in our study. However, the possibility of the occurrence of the organism in this state in study area has to be considered in future studies, to generate comprehensive data on the occurrence and persistence of *V. cholerae* in study area.

*V. cholerae* O1 co-existed with non-O1strains in some streams and wells (Additional file 1). Horizontal gene transfer during co-existence of O1 and non-O1/non-O139 strains [29] has been reported to result in the emergence of novel pathogenic serogroups as well conversion of non-toxigenic strains to toxigenic strains increasing the concentration of toxigenic *V. cholerae* in water and the possibility of an outbreak of cholera.

*Vibrio cholerae* O139 was not detected during our study. Cholera outbreaks in Africa have been caused by the O1 serogroup. There are no reports of cases due to the O139 infection as this serogroup is confined to Southeast Asia. *V. cholerae* O1 was isolated only from stream (47.1%, 8/17) and well (34.0%, 17/50) samples (Tables 1, 2; Additional file 1) confirming these water sources as reservoirs of the organism in study area. Streams studied are used as dump sites for human and domestic waste. In addition, open drains which carry human wastes empty into these streams. This may explain the high prevalence of the organism in streams. Based on our findings, the use of streams for bathing, recreation and irrigation should be discouraged as this could result in health hazards. The majority of wells were unprotected and due to overcrowding they are located at close proximity to sanitary infrastructures. See page from sanitary facilities through the porous and sandy soil of study area [30] could result in contamination of wells. The only contaminated tap water sample contained *Vibrio cholerae* non-O1. Municipal water receives adequate treatment before distribution. Contamination could have occurred post treatment through a broken pipe along the distribution network.

Although the rate of isolation of O1 was slightly higher in the rainy season (19/49, 38.8%) than in the dry season (6/19, 31.6) (Table 2), the difference was not significant (χ^2^=0.0.305, P=0.581). Most parts of study area usually experience floods after heavy rainfall. Flood waters distribute infectious agents from sanitary infrastructures into streams and wells (as majority are poorly constructed) thus contaminating water from these sources. Isolation of the organism in both seasons and in all the months of our study (Table 2, Additional file 2) indicates its presence all year round in study area.

Susceptibility of *V. cholerae* O1 to antibiotics previously used in prophylaxis and cholera treatment in Douala [31,32] as well as other recommended antibiotics for cholera treatment [33] was analyzed to determine the most appropriate agents for disease management to reduce case fatality rate in the event of an outbreak. Although findings of present study show 100% susceptibility to ciprofloxacin, there are reports of emergence of ciprofloxacin resistance in other parts of the world [10]. Other drugs with high susceptibility were chloramphenicol (80%) and doxycycline (88%) (Additional file 3). Previous studies [32] have also reported high susceptibility of clinical isolates from Douala to these drugs. Our findings thus validate their use in cholera treatment and prophylaxis in Douala. However, to preserve the high potency of ciprofloxacin, doxycycline and chloramphenicol could be used as alternative therapeutic agents.

Resistance to β-lactams (92% for ampicillin and 88% for amoxicillin) as well as tetracycline (68%) and cotrimoxazole (64%) was high (Additional file 3). Tatah *et al.*[34] also reported low susceptibility of *V. cholerae* O1 and non-O1isolates from another cholera endemic locality in Douala to these drugs. The association between development of resistance and large scale use of these agents for cholera treatment and prophylaxis in Douala is well recognized [9,16]. Resistance to these agents has been reported in other developing countries [10]. These antibiotics are also being used extensively or misused for the treatment of other infectious conditions present in study area other than cholera and could have selected for resistant strains in study area. However, strictly prescribed and controlled use of antibiotics for prolonged periods may not affect susceptibility profiles of *Vibrio cholerae*[31,35]. Although drug resistance is not a virulence factor, it may play a role in the selection, persistence and dissemination of pathogenic strains of *V. cholerae* which are difficult to eradicate. Antibiotic resistance has influenced change in policy on cholera treatment in some countries [36] indicating the relevance of knowledge on current susceptibility patterns of pathogens. Our findings highlight the need for constant evaluation of antibiotic susceptibility pattern of *V. cholerae* particularly as it is persistent in New Bell, to understand its epidemiologic features. We tested for antibiotic susceptibility of isolates using the disc diffusion technique. MIC of antibiotics analyzed was not determined. This constitutes a limitation to our study.

The emergence of resistance to various antibiotics among vibrios is a well established phenomenon. Isolates in our study showed a heterogeneous antibiotic resistance pattern with 9 multi-drug resistance (MDR) patterns detected among isolates showing MDR (Table 3). Other studies [37] have reported a lower frequency of multidrug resistant *V. cholerae* than observed in our study. The two isolates (from W12 and W68) not showing multidrug resistance, were resistant to doxycycline but sensitive to chloramphenicol. WHO recommends the use of doxycycline or ciprofloxacin as treatment choice for cholera [38]. Ngandjio *et al.*[9] reported multidrug resistance in all isolates obtained during the 2004–2005 cholera epidemic in Douala. These multidrug resistant strains could have been disseminated into the environment resulting in the high levels reported in our study. Multidrug resistance limits the use of these agents for empiric cholera treatment. Although little is known about the antibiotic susceptibility of epidemic *V. cholerae* strains particularly in African countries, our results indicate that they could pose a public health threat in study area. The high level of multi-drug resistant strains reported in this study and in previous reports from Douala [9,16] indicates the possibility of mobilization of resistance markers among isolates and calls for further studies on plasmid profiles to analyze for the presence of plasmids such as the SXT element, Class 1 integrons [39], transposoons [40] (which have been reported to confer resistance in vibrios) as mechanisms of acquisition of drug resistance genes in our isolates. Since the emergence of such resistance among *V. cholerae* may significantly influence future strategies for cholera control, continuous monitoring of epidemic strains is thus crucial.

Although the majority of environmental *V. cholerae* strains are considered harmless, strains have evolved that cause disease in humans by effectively colonizing the small intestine and releasing potent enterotoxins. To determine the public health significance of isolates, we analyzed for the presence of *ctx*A and *tcp*A, which are the major virulence markers of *V. cholerae*; and *zot*, a supplemental pathogenic factor (Table 4). PCR analysis revealed 28% (7/25) of isolates being toxigenic strains. The rest were negative for 302bp *ctx* indicating that these strains do not have the genetic potential to produce cholera toxin. These results are in agreement with the findings of Chakraborty *et al*. [41] and Alam *et al*. [13] who reported a low prevalence of toxigenic *Vibrio cholerae* in environmental samples. This may indicate a better adaptability of non-toxigenic *V. cholerae* over toxigenic strains in water. The El Tor hemolysin has been shown to be the virulence factor responsible for development of diarrhea in non-toxigenic strains [42] making them a public health concern. Cholera toxin production has been detected in CT-positive organisms [43] implying that there is no silent CT gene. Although we did not analyze *ctx*-positive isolates for disease potential, it is important for them to be considered as pathogenic since cholera enterotoxin is a major virulence factor.

Fifteen isolates (60%) had genes encoding for the A subunit of toxin co-regulated pilus (TCP) of which 13 had *tcpA* of the El Tor biotype and 2 positive for *tcpA* of the Classical biotype. Only four isolates positive for *ctx* gene were also *tcpA* positive. Eleven (73.3%) *tcpA* positive isolates were negative for the *ctx* gene. Our finding is contrary to the general notion that most *ctx* positive strains are also positive for *tcp*. Possession of *tcpA* therefore suggests that these strains are potentially pathogenic since they have the ability to colonize the human intestine and are susceptible to conversion to toxigenic strains by CTXΦ either inside the host intestine or in the aquatic environment. Such *V. cholerae* with latent pathogenic potential have been reported in aquatic environment in other countries [41,44]. Two were positive for *tcpA* Classical gene and the rest two positive for *tcpA*_*ET*_. All *tcpA*_*CL*_ positive isolates were toxigenic. This is the first report on the presence of the *V. cholerae* with Classical type *tcpA* in Douala. This finding is of great significance and suggests the possibility of the emergence of strains of the Classical biotype in study area.

Zonular occludens toxin gene was detected in 15 (60%) isolates indicating a high prevalence of *zot* in isolates (Table 5). Eleven of these were non-toxigenic. The *zot* gene encodes zonular occludens toxin (*zot*) described by Fasano *et al.*[45] as a toxin that increases the permeability of the small intestinal mucosa by affecting the structure of the intercellular tight junctions. Our findings contradict the results of Rivera *et al*. [24] who reported the absence of *zot* in non-toxigenic *V. cholerae* O1. Toxigenic *V. cholerae* have been shown to contain a compound transposoon-like structure, the CTX genetic element which comprises a core region that contains the cholera toxin A and B subunits (*ctx*AB), zonular occludens toxin (*zot*), accessory colonization enterotoxin (*ace*), an open-reading frame of unknown function (*orf*U) and core-ended pilus (*cep*) genes. Thus the *zot* gene is therefore expected to present in toxigenic *V. cholerae* strains likewise the *ctx* gene in *zot*-positive strains. However, Ghosh *et al.*[46] and Jiang *et al.*[47] reported the presence of *zot* gene in non-toxigenic strains, confirming our findings. These findings contradict earlier reports [48] showing that the *zot* gene does not occur independently of the *ctx* genes and as such cannot be used to explain the ability of some *V. cholerae* strains to cause illness in the absence of cholera toxin. Findings of our study indicate the possibility of the CTXΦ prophage genome missing or disrupted by mutation, meaning that many CTXΦ genes among these strains are likely defective, a phenomenon commonly found among all genera of bacteria. However, of the 15 *zot*-positive isolates, 7 (28%) contained only *zot* and 8 isolates carried the *zot* and other virulence factors.

Based on the genes investigated, 7 genotypes were observed among *V. cholerae* O1 isolates with genotypes *ctx*A^-^*tcp*A_ET_^+^*zot*^-^ (28%) and *ctx*A^-^*tcp*A_ET_^-^*zot*^+^ (28%) predominating. Apart from recording a great diversity of genotypes in study area, we also detected the presence of more than one genotype from same source. Isolates with the genotype *ctx*^*+*^*tcpA*_*ET*_^*-*^*zot*^*+*^ and *ctx*^*+*^*tcpA*_*ET*_^*-*^*zot*^*-*^ (Table 5) were toxigenic but negative for the *tcpA*. This suggests the presence of a tcp-independent mechanism for infection of these isolates by CTXΦ [49,50]. These genes studied are located on mobile genetic elements and could be transferred to avirulent strains. However, there was a high prevalence of toxigenic and potentially toxigenic genotypes among isolates.

Sample collection for our study started in March 2010 and by May 2010, an epidemic of cholera was declared in Douala. This may explain the high prevalence of toxigenic *V. cholerae* O1 compared to previous reports [51]. Analysis of DNA fingerprints is necessary to show a link between isolates from present study with those from the 2010–2011 epidemic. Our findings show the necessity for surveillance of *V. cholerae* in aquatic environments in cholera endemic areas of Cameroon to facilitate disease prediction, prevention and control so as to avert the devastating consequences of an epidemic.

The dynamics *V. cholerae* has been shown to be influenced by environmental factors [28,52] through a shift in pathogen or host reservoir species abundance, population dynamics, and community interactions [53]. The influence of environmental factors: temperature, pH and salinity on occurrence of *V. cholerae* O1was investigated (Additional file 4)*.* Temperature of samples ranged from 22.8°C to 31.0°C in the rainy season and 26.9°C to 33.3°C during the dry season. There was no significant difference in stream water temperatures between the rainy and dry season (Z = −1.389; with P > 0.05). However, there was a significant variation in temperature of well water samples between the two seasons (P = 0.001). There was no correlation between temperature and occurrence of *V. cholerae* O1 in the various water sources in the dry season (Spearman’s rho = −0.069, P = 0.454) and in the rainy season (Spearman’s rho = −0.019, P = 0.804). Aulet *et al*. [54] isolated *V. cholerae* in water sources with temperatures ranging from 15°C to 26°C with optimal isolation at 25°C. This falls within the temperature range of samples collected during the rainy season in which the recovery rate was higher. Although some studies [55] have reported no correlation in isolation pattern of *V. cholerae* and maximum temperature recorded others [56,57] have reported temperature to correlate with the occurrence of *V. cholerae*, hence an important modulator of environmental concentrations of vibrios. Our study and the report of Dalsgaard *et al*. [58] showed no correlation between temperature and the isolation of *V. cholerae.* A study with longer duration of sampling in our study area will permit valid conclusions about the influence of temperature on the occurrence of *V. cholerae.*

pH values ranged from 5.7 to 9.1 in the rainy season and 6.3 to 11.3 in the dry season. There were significant differences in the pH values of stream and well samples between the rainy and dry season (Z = −3.748; Z = −3.531 with P < 0.05 for stream and well respectively). There was no significant difference in tap water values between the two seasons (Z = −1.838, P = 0.066). Optimal pH for isolation of *V. cholerae* O1 has been reported to vary between 7.0 and 8.5 and the organism is inactivated at pH below 4.5 [59]. pH values reported in this study are similar to the optimal pH of 8.5 reported by Huq *et al*. [60] for attachment and multiplication of *V. cholerae* on copepods. There was a significant correlation between pH and occurrence of *V. cholerae* O1 in the various water sources in the dry season (Spearman’s rho = 0.227, P = 0.013) and the rainy season (Spearman’s rho = 0.380, P = 0.0001). Our findings contradict the results of Blackwell and Oliver, [57] who reported pH to have no significant correlation with the isolation of *V. cholerae* in water samples from a major shrimp production area in Thailand.

Salinity ranged between 1.62 and 11.03 ppt in the rainy season and 0.57 and 5.16 ppt in the dry season. There was a significant correlation between salinity and occurrence of *V. cholerae* in the various water sources in the dry season (Spearman’s rho = 0.201, P = 0.003) and the rainy season (Spearman’s rho = 0.280, P = 0.0011). Salinity has been demonstrated to influence significantly the growth of *Vibrio cholerae* in cholera endemic areas [14,47]. Salinity values in present study fall within the range for detection of *V. cholerae* (between 2 and 14 ppt, with higher recovery at values below 8 ppt) reported by Louis *et al.*[14]. Jiang [47] detected higher concentrations of *V. cholerae* at salinities below 10 ppt but above 0 ppt. Huq *et al*. [58] reported salinity alone not having an influence on growth of *V. cholerae* at temperatures as low as 10°C while at higher temperature the influence of salinity was significant.

## Conclusion

Our results thus demonstrate the presence of diverse genotypes of multidrug resistant toxigenic and potentially toxigenic *V. cholerae* O1 in New Bell. This study has also demonstrated the presence of toxigenic strains with classical type *tcpA*. Salinity and pH are some of the factors that could be maintaining its occurrence and persistence of the pathogen in study area. Based on our findings, we recommend the expansion of potable water distribution in New Bell, provision of appropriate sanitary infrastructures, routine well inspection and disinfection as measures for disease prevention in study area.

## Abbreviations

CDC: Center for disease control and prevention; MINSANTE: Ministere de la sante publique (Ministry of public health).

## Competing interests

The authors declare that they have no competing interests.

## Authors’ contributions

JTKA as principal investigator conceived, designed and coordinated the study, interpreted data and initiated the writing of the manuscript. TNM collected samples, isolated and characterized bacteria carried out antimicrobial susceptibility testing and together with HAN carried out molecular studies. All authors read and approved the final manuscript.

## Pre-publication history

The pre-publication history for this paper can be accessed here:

http://www.biomedcentral.com/1471-2334/13/366/prepub

## Supplementary Material

Additional file 1 **Distribution of *Vibrio cholerae *O1 in various water sources.** Click here for file

Additional file 2 **Isolation of *V. cholerae *O1 strains by month.** Click here for file

Additional file 3 **Antibiotic susceptibility of *Vibrio cholerae *O1 isolates.** Click here for file

Additional file 4 Physico-chemical characteristics of water samples. Click here for file
